# The Role of Iron and Cobalt in Gynecological Diseases

**DOI:** 10.3390/cells12010117

**Published:** 2022-12-28

**Authors:** Adrianna Ćwiertnia, Mateusz Kozłowski, Aneta Cymbaluk-Płoska

**Affiliations:** Department of Reconstructive Surgery and Gynecological Oncology, Pomeranian Medical University in Szczecin, Al. Powstańców Wielkopolskich 72, 70-111 Szczecin, Poland

**Keywords:** iron, cobalt, ovarian cancer, endometrial cancer, cervical cancer, uterine myoma, endometriosis, polycystic ovary syndrome, placental hypoxia, radiotherapy

## Abstract

Iron and cobalt are micronutrients that play an important role in the regulation of cellular processes, being part of the centre of catalases, peroxidases, cytochromes and metalloproteins such as hemoglobin and myoglobin (Fe). Cobalt primarily functions as a component of hydroxycobalamin, which is essential for regulating red blood cell production. Maintaining normal levels of cobalt and iron in the human body is important, as a deficiency can lead to anaemia. These elements are also involved in reactions during which oxidative stress occurs and are therefore considered to be a cause of tumor formation. This paper will discuss aspects of the influence of cobalt and iron on mechanisms that may contribute to the growth of gynecological tumors, as well as other obstetric-gynecological disease entities, by altering the conditions of the microenvironment. In addition, the following review also highlights the role of cobalt and iron in the treatment of gynecological tumors.

## 1. Introduction

Iron and cobalt are among the micronutrients that have important functions for maintaining normal homeostasis in the human body [1,2]. Among other things, iron is involved in oxygen transport as a component of hemoglobin [3], and cobalt is embedded in the ring of hydroxycobalamin (vitamin B12), ref. [4] which is involved in erythropoiesis [5]. Iron and cobalt deficiency can lead to microcytic [6] and macrocytic [7] anaemia, respectively. During pregnancy, there is an increased iron requirement of approximately 1000–1200 mg, of which 1/3 of this iron pool is needed for placental-fetal tissues [8]. The causes and consequences of deficiencies of these micronutrients are well understood and described in the literature. The second equally important issue concerning cobalt and iron, is their excess. Their toxicity has been studied for several decades, as well as the links between cobalt and iron overload and tumorigenesis.

Cobalt and iron are among the elements that compete with each other, due to the fact that they most commonly occur on the same oxidation levels (2+/3+) [9] and the fact that they have similar properties [10]. Iron is a well-documented carcinogen in both animals and humans [11]. In contrast, there are no conclusive scientific reports on the potential carcinogenic effects of cobalt, and the evidence is more based on animal cell studies. However, cobalt metal and soluble cobalt(II) salts belong to the group of carcinogens for which there is evidence from studies with human cells and can be considered likely [12].

The most common gynecological malignancies are endometrial cancer, cervical cancer and ovarian cancer [13]. Ovarian cancer is the most common cause of death among patients with gynecological malignancies [14]. Very often its first symptoms appear already at an advanced stage [15]. In the prevention of cervical cancer, human papillomavirus (HPV) vaccines play a special role [16]. In contrast to ovarian cancer, endometrial cancer more often manifests itself at an early stage [17]. Symptoms of endometrial cancer mainly include abnormal uterine bleeding [18]. Hysteroscopy with histopathological examination allows initial diagnosis and initiation of treatment [19]. Diseases such as polycystic ovary syndrome and endometriosis, which increase the risk of endometrial cancer, or ovarian cancer, may also be associated with tumorigenesis [20].

The following literature review underline the role of cobalt and iron in gynecological diseases, folliculogenesis, placental function, as well as the effects of excess of these micronutrients on the development of gynecological cancers and the use of the radioisotope cobalt Co-60 in radiotherapy. We also discuss processes and reactions involving both elements, including ferroptosis as non-apoptotic cell death, which may prove useful in cancer therapy. The review of the work relates to the state of knowledge up to November 2022.

## 2. The Role of Iron

### 2.1. Iron

Iron is a micronutrient that belongs to the transition metals and has a number of important functions in the human body. First and foremost, it is essential for oxygen transport by hemoglobin, with which more iron is associated in the body [21]. Iron is used in a number of reactions due to its two degrees of oxidation: heme iron Fe^2+^ (ferrous cation) and non-heme iron Fe^3+^ (ferric cation) [22]. Reduction of Fe^3+^ to Fe^2+^ occurs by duodenal cytochrome b (Dcytb) and can then be transported into duodenal mucosal cells by the divalent metal transporter (DMT1) [23]. Fe^2+^ in the presence of superoxide and hydrogen peroxide (H_2_O_2_) can catalyse the formation of hydroxyl radical (*OH), and this, due to its oxidative properties, promotes lipid peroxidation, mutagenesis and DNA strand damage, oncogene activation and suppression of suppressor genes [24]. These two steps enable redox reactions to occur and thus the ability of various ligands to bind iron. Performing a number of biologically relevant functions, it also involves the generation of reactive oxygen species (ROS), which takes place during the Fenton reaction [25,26]. This occurs when superoxide (O_2_*-) releases ferric iron (Fe^3+^) from ferritin and hemosiderin in the cell [24]. Iron may affect the formation of lipofuscin, one of the better understood markers of ageing. Moreover, free radical formation via lipofuscin can be four times higher with iron than without it [27].

### 2.2. Transferrin

Transferrin (Tf), which is a glycoprotein that binds iron in the form of Fe^3+^ (ferric iron), is responsible for the binding and transport of iron absorbed from the gastrointestinal tract into tissues [28]. Thanks to transferrin receptors (TfR1) on the surface of cells, which are involved in iron uptake, there is no excessive transport of iron into the cells [29]. However, small amounts of iron can also be bound to ferritin as a hemopexin-hem complex and haptoglobin-hemoglobin, which is particularly important in cases of iron overload. Non-transferrin-bound (NTB) forms are directed to the liver, stored as ferritin or re-transported to plasma to bind to transferrin [30]. To maintain normal iron homeostasis, transferrin receptor 2 (TfR2) is responsible for the expression of hepcidin and regulates erythropoiesis, being located on erythroid precursors as a component of the erythropoietin (Epo)-Epo receptor (EpoR) complex [31]. Levels of iron and the molecules responsible for its transport, storage and metabolism should be tightly regulated, as increased iron stores as well as transferrin are associated with free radical formation [32].

### 2.3. Ferroportin

Ferroportin is a trans-membrane protein that acts as an iron transporter (iron efflux pump). It allows the absorption of iron in enterocytes (transcytosis). Ferroportin is also found in hepatocytes, spleen, kidney, Kupffer cells and macrophages [33]. Its activity can be regulated by transcription and also post-translationally. Iron deficiency, hypoxia, transition metals, hem and inflammatory cytokines regulate ferroportin transcription. Importantly, ferroportin is influenced by hepcidin, a peptide hormone that causes the internalisation and degradation of ferroportin [34].

### 2.4. Ferroptosis

Ferroptosis is a type of cell death that occurs due to the accumulation of reactive oxygen species, which is iron-dependent [35]. Ferroptosis is not associated with either apoptosis or cell necrosis [36,37]. Doubts remain about autophagy, with which ferroptosis may have more in common. This is due to the accumulation of autophagosomes in response to ferroptosis-inducing factors. More specifically, the proteins involved in autophagy ATG3, ATG5, ATG4B, ATG7, ATG13 and BECN1 may also contribute to cell death by ferroptosis [38]. In its course, lipid hydroperoxides (LOOH) and iron ion (Fe^2+^) concentrations are increased in cells. Excessive amounts of reactive oxygen species initiate lipid peroxidation via Fenton reactions. This is followed by damage to the outer mitochondrial membrane, its rupture and condensation [37]. The process of ferroptosis can be initiated by biological or chemical agents, and the pathways leading to this cellular destruction are divided into extrinsic (transporter) and intrinsic (enzymamtic) [39,40]. The activating factor for ferroptosis may be signal transducer and activator of transcription 3 (STAT3), as demonstrated in pancreatic ductal adenocarcinoma cell lines [41]. Ferroptosis can also be promoted by ferritinophagy involving nuclear receptor coactivator 4 (NCOA4), Ras-related protein Rab-7a (RAB7A)-dependent lipophagy, inhibition of the xc system by Beclin 1 (BECN1) and the aforementioned autophagy together with chaperones [38]. Most importantly, the p53 protein reduces cystine uptake by inhibiting the expression of the cystine transporter Solute Carrier Family 7 Member 11 (SLC7A11) and sensitises cells to ferroptosis [42]. Enhancement of ferroptosis can also occur by increasing the expression of spermidine/spermine-N-acetyltransferase 1 (SAT1) and glutaminase (GLS1) [43]. However, p53 can also inhibit ferroptosis through direct inhibition of dipeptidyl peptidase 4 (DPP4) activity or through induction of cyclin-dependent kinase inhibitor 1A (CDKN1A/p21) [44]. In addition, it is possible that ferroptosis affects the immune properties of the tumor [45]. Ferroptosis therapies are being considered as a potential treatment for refractory triple-negative breast cancer, whose cells show sensitivity to ferroptosis-stimulating agents [46]. Ferroptosis-inducing drugs and iron chelating agents are being considered as effective cancer treatments. Used together with chemotherapy, radiotherapy and immunotherapy, but also after taking into account that different tumor cells may have different sensitivities to iron-induced death, they may have significant therapeutic value [47].

### 2.5. Iron and Carcinogenesis

Carcinogenesis is promoted by the formation of reactive oxygen species, which can lead to DNA and protein damage. This results in mutation of proto-oncogenes and tumor suppressor genes [48]. The effect of iron on tumorigenesis is well documented on animal cells, although there are no clear conclusions in all studies. Iron as an agent of carcinogenesis has been particularly studied on lung, kidney and peritoneal cells [49]. Some studies confirm that excess iron makes tumors grow faster [50]. Furthermore, it is possible that reduced iron concentrations in premenopausal women and blood donors, as well as hepatitis C patients who underwent phlebotomies, are associated with a lower risk of tumorigenesis [51]. Animal cell studies suggest that iron deficiency may also contribute to a higher incidence of neoplastic proliferation compared to groups with normal iron levels [52]. However, observations of lung adenocarcinoma proliferation in mice show that iron loading does not affect tumor development and that tumor size is also not dependent on iron concentration [53]. An explanation for the abnormal iron metabolism in tumour cells can be explained by the example of renal cell transformation, in which ferritin is downregulated by transforming protein p21 (H-Ras). This leads to increased levels of labile iron ions, and these lead to tumour cell proliferation [54].

## 3. The Role of Cobalt

### 3.1. Cobalt

For humans, the main source of cobalt is animal food [55], indicating a risk of vitamin B12 deficiency in vegetarians [56]. In serum, cobalt ions (Co(^2+^)) are bound to albumin and are subsequently incorporated into vitamin B12 [57]. The binding of cobalt to this vitamin is its key role in the human body [58]. Vitamin B12 is a hydroxycobalamin, and due to the hemin moiety into which cobalt is incorporated, this vitamin is not toxic to humans [59]. Cobalamin functions as a cofactor for two enzymes: methionine synthase and methylmalonyl-CoA mutase [60]. Vitamin B12 deficiency can result in neurological and hematological disorders and may also be a risk factor for coronary heart disease [61]. In contrast, excess hydroxycobalamin can lead to liver disease, malignancies, including hematological malignancies [62]. Controlling vitamin B12 levels is possible by determining serum vitamin B12, checking serum homocysteine, holotranscobalamin II or methylmalonic acid levels [56]. Cobalt compounds may be present and contribute to people’s daily lives. This is shown in a study by Cheong et. who showed that the release of cobalt from jewellery and metal clothing items is high [63]. The use of cobalt in industry carries a risk of developing occupational diseases, which occurs as a result of inhaling its compounds. In the past, there have been exposures to cobalt chloride in tablet form, which was used to treat anemia [64]. It was withdrawn due to its toxicity. Cobalt has also been used in the production of beers. The increase in the number of cardiomyopathies has been linked specifically to the consumption of cobalt, because shortly after it was removed from brewery production, no equal or greater incidence of heart disease was observed [65]. There are a number of methods that allow the use of biological indicators of cobalt exposure such as cobaltemia and cobalturia. This allows toxicological testing, which is particularly useful in groups exposed to cobalt-containing dusts [66].

In cells, cobalt exists as a divalent or trivalent cation, where it combines with other molecules. This results in the formation of oxides, chlorides or other cobalt compounds [67]. Through its similarity to iron (II) ions, cobalt can occupy the Fe^2+^ site in the heme porphyrin ring, resulting in a deoxygenated form of the protein. This results in hypoxia, whereby tissues are hypoxic, and the erythropoietin gene is activated in response [60]. It is possible that there is a synergistic toxicity of cobalt and zinc [68]. In addition to porphyrin, cobalt cations may enter enzyme sites instead of zinc, magnesium or manganese ions. This is associated with lower activity of these enzymes [69]. Moreover, they inhibit RNA polymerase [70]. Furthermore, excess cobalt can lead to hyperglycemia in animals, which happens through damage to pancreatic alpha cells [71]. The negative effects of cobalt on lung cells have also been described. Its toxicity may depend on the solubility of the cobalt compound. According to Smith et al. soluble cobalt compounds are more cytotoxic to human lung fibroblasts than insoluble ones [72]. There are also reports that cobalt may contribute to the development of pneumoconiosis and asthma [71]. Radioactive cobalt (60)Co and (58)Co can be a dangerous source of radiation for hospital workers [73].

### 3.2. Cobalt Chloride

Cobalt chloride is primarily used as an additive in feed and fertilisers [74,75]. The literature describes that cobalt salts contribute to the induction of mutagenic agents by inhibiting DNA repair [76]. Compared to other cobalt compounds, CoCl_2_ shows higher toxicity, and the toxicity of cobalt is linked to its solubility. More specifically, Co3O4 is a compound that is 99% excreted in faeces, is not soluble in water and is not a highly toxic compound compared to others. In contrast, 80% of cobalt (II) chloride excreted in feces is water soluble [77]. The relationship between the toxicity of cobalt and its solubility was also investigated by Smith et al. Conducting a study on human lung fibroblasts, it was noted that soluble cobalt may be more cytotoxic than solid cobalt. However, genotoxicity in this study was at comparable levels in both cobalt states. Furthermore, soluble cobalt contributes to cell cycle arrest at lower intracellular concentrations than cobalt oxide [72]. CoCl_2_ is known as a compound that can lead to hypoxia-like conditions in the cell. Such processes result from the production of reactive oxygen species [78] in a non-enzymatic, non-mitochondrial mechanism [79]. In the presence of CoCl_2_, hypoxia-inducible factor (HIF-1α), which has a role in angiogenesis, is activated [80]. Through increased cobalt-induced HIF-1α levels, increased erythropoietin production and thus erythropoiesis is also observed [79]. This means that the HIF-1α protein cooperates with genes encoding angiopoietin, erythropoietin and, moreover, also with genes encoding vascular endothelial growth factor, insulin growth factor and also proapoptotic proteins (for example, proetin encoded by the stress-responsive gene DNA-damage-inducible transcript 4 (RTP801) and protein related to the BH3-only family NIX) [81]. In addition to other factors, it is hypoxia conditions that may contribute to cancer metastasis, which particularly emphasises the importance of a thorough understanding of the properties of this compound. Under hypoxic conditions, HIF-1α binds to nuclear b-catenin, which enhances their action. It has been suggested that transcriptional associations with HIF-1α and NF-kB, β-catenin/p300 are able to alter tumor cell kinetics [82].

### 3.3. Cobalt and Cancerogenesis

For several decades, attempts have been made to resolve whether cobalt and its compounds are teratogenic (Table 1). It is known that cobalt is a potent initiator of oxidative stress in cells, thereby altering gene function and consequently may increase the risk of cancer [83]. 30 years ago, single experiments were based on the possibility that cobalt could cause cancer, but conclusions were made based on observations of the effect of cobalt on cells only at the site of injection. It was not possible to say unequivocally that cobalt was a factor in carcinogenesis [58]. For example, cobalt alloys are present in orthopedic implants. However, a meta-analysis testing whether they contribute to an increased risk of cancer did not show a link between the above [84,85]. A review of the literature on cobalt as a potential carcinogen shows that the evidence is mainly based on animal cell studies. Furthermore, when looking at cobalt, compounds of this metal should also be considered. Cobalt can exist as cobalt (II) and cobalt (III), with cobalt in the second oxidation state more commonly used in the chemical industry [10]. The properties of cobalt in ionic form differ from those of cobalt compounds. The International Agency for Research on Cancer have provided a classification of cobalt and its compounds into appropriate groups according to tumorigenic potential. It appears that cobalt without other metal alloys and soluble cobalt (II) salts can be classified as probably carcinogenic to humans, placing them in group 2A. Cobalt (II) oxide belongs to group 2B, indicating possibly carcinogenicity in humans. In contrast, cobalt (II,II) oxide, cobalt (II) sulfide and other cobalt (II) compounds belong to group 3 and are not classified as carcinogenic to humans [12]. A particular cobalt compound to look at is CoCl_2_. It induces hypoxia, which leads to inhibition of proliferation of a human ovarian cancer cell line [86].

### 3.4. The Role of Cobalt in Cancer Therapy

Treatment of cervical cancer stage IB2 and higher according to FIGO consists of chemo-radiotherapy with brachytherapy [87]. In the treatment of advanced cancers, radiotherapy in combination with cisplatin is used after surgery [88].

Radiotherapy can be distinguished by high-dose rate (HDR) brachytherapy [89]. Brachytherapy allows a high dose of radiation to be delivered to the tumor with limited radiation reaching normal tissues that are close to the tumor [90]. In the case of gynecological tumors, this will mainly be the bladder and rectum [89]. The main radioisotopes used in radiotherapy of cervical cancers are Ir-192 and Co-60, with iridium being the more commonly used isotope [91]. A compilation of results showing the toxicity and impact on patient survival depending on the radioisotope used is an important subject of research. Some sources show that Ir-192 and Co-60 do not differ in terms of survival or toxicity when used in brachytherapy [91,92]. A study importing the effects of iridium and cobalt radiation on the bladder and rectum showed no differences between the two isotopes [93]. Other work presents iridium as a radioisotope with better physical properties, allowing for more satisfactory brachytherapy effects [94]. It is likely that Ir-192 may contribute to greater tumor regression and will also damage nearby tissues to a lesser extent [95]. It is possible that cobalt-60 is most effective in low-grade tumors and those less than 2 cm in diameter [96]. An important consideration in the choice of radioisotope for the treatment of gynecological cancers is the cost of therapy. The cobalt isotope compares more favourably in economic terms [92]. This is related to the longer half-life of the cobalt isotope than that of iridium, 5.26 years, 73.8 days respectively, which contributes to the need to replace the iridium source 3–4 times per year [97]. Even the need for larger shielding when using cobalt than when using iridium brachytherapy does not outweigh the cost of using Ir-192 [98]. Therefore, brachytherapy with Co-60 is proving to be an important alternative for hospitals with lower financial resources [99]. Furthermore, according to the examples in the literature, it can be noted that cobalt compounds differ in their biochemical and biophysical properties. These characteristics and the lower toxicity of cobalt compared to other transition metals have sparked interest in cobalt as a new potential anticancer drug that could yield clinically relevant results [100]. Pasukonien created cobalt ferrite nanoparticles (Co-SPIONs) to study uptake, toxicity and effects on stem-like properties in the human ovarian cancer cell line A2780 and pancreatic cancer cell line MiaPaCa2. It was observed that both cell lines accumulated Co-SPIONs, but that A2780 cells (ovarian cancer cell line) were more sensitive to cobalt ferrite exposure. The nanoparticle could be used for diagnostics and targeted cancer therapy, but the safe concentration should be carefully assessed depending on the type of cancer cells [101]. Furthermore, it has been shown that three cobalt (II) complexes [Co(MQL)2Cl2] (CoCl_2_), [Co(MQL)2Br2] (CoBr) and [Co(MQL)2I2] (CoI), containing 8-methoxyquinoline (MQL), can probably exhibit greater antiproliferative activity than cisplatin for the treatment of cisplatin-resistant ovarian cancers [102]. The work by Law et al. tested whether six different cobalt tris(bipyridine) complexes, depending on a given concentration, could inhibit the proliferation of cancer cells in various tissues, which would occur by arresting cell cycle progression. In the case of cervical cancer cells (HeLa) to which complex 2 [CoIII(4,4′-Me2-bpy)3](PF6)3 was applied, only a high concentration stopped the HeLa cancer cell in S phase. To confirm this, the levels of cell cycle regulatory proteins from G1 to S phase were examined. An accumulation of cyclin A and E2F was observed, which could suggest that complex 2 prevents the proliferation of the cancer cell by arresting it in S phase. This study demonstrates the possible efficacy of alternative metal compounds in cancer cells in vivo in arresting their proliferation, which is important data, especially considering the limitations of cisplatin, carboplatin in cancer therapy due to the production of resistance against these compounds after the initial treatment [103].

## 4. Iron and Cobalt in Gynecological Diseases

### 4.1. Ovarian Cancer

**Figure 1:**
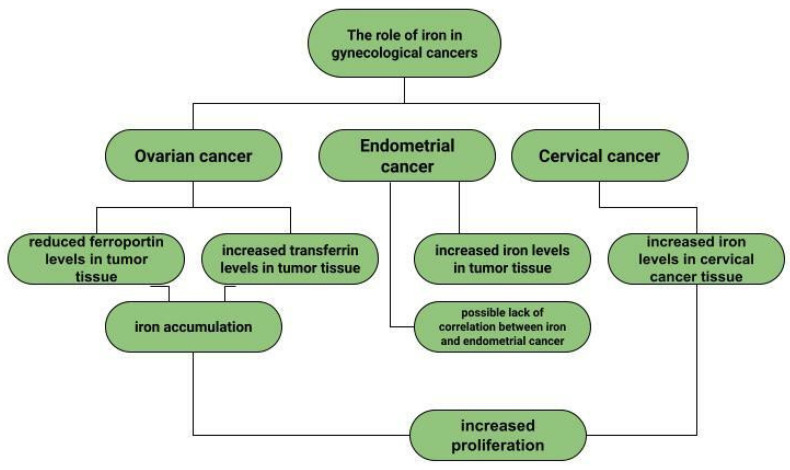
The role of iron in gynecological diseases.

**Figure 2:**
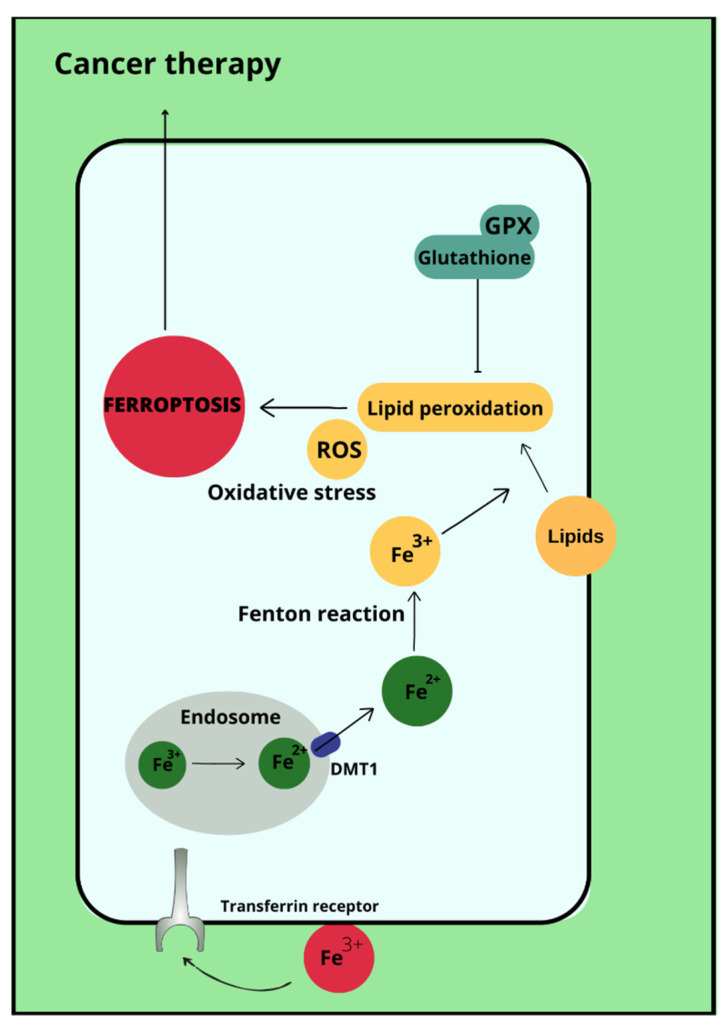
Ferroptosis in cancer therapy.

Ovarian cancer is a neoplasm that often manifests itself only at a late stage [104], and symptoms are often non-specific, sometimes resembling gastrointestinal symptoms [105]. Among other things, late diagnosis contributes to ovarian cancer being the most common cause of death among gynecological cancer patients [14]. Risk factors for ovarian cancer include early menarche, late menopause [106], BRCA1 and BRCA2 gene mutations [107], obesity [108], and asbestos [109] and talc [110] exposures. In addition to the above, the effect of metals on ovarian cancer risk is also being investigated. In ovarian cancer tissues compared to non-malignant tissues, a higher iron content in the second oxidation state is observed. In non-malignant lesions, such as in ovarian cyst fluid, the greater proportion of iron is Fe^3+^ [111]. These observations are not confirmed by the Yaman et al. study, in which no difference in iron concentrations was observed for cancerous and non-cancerous tissues [112]. Another study examined what effect a reduction in intracellular iron concentration has on tumor growth. The results led to the conclusion that ovarian cancer cells are sensitive to ferroptosis and iron chelators, which could be used in ovarian cancer therapy [113]. Basuli shows that ferroportin (FPN) levels are reduced in tumor tissue of high-grade serous ovarian cancer. In contrast, transferrin (TFR1), an iron importer, is increased in this tissue. This leads to iron accumulation in the tissue and increased proliferation (Figure 1) [113]. Dan Sun et al. examined the function of lidocaine in the process of ferroptosis of ovarian and breast cancer cells. They reported that lidocaine inhibited mRNA expression of solute carrier family 7 member 11 (SLC7A11) in a dose-dependent manner-3mM lidocaine produced the highest effect. Co-treatment of lidocaine and erastin was able to increase the effect of erastin on inhibiting ovarian cancer target line SKOV-3 and T47D. Total iron and ferrous iron (Fe^2+^) were analysed in the cells. Fe^2+^ levels were upregulated by lidocaine in SKOV-3 and T47D cells. This study also noted that lidocaine promoted lipid ROS accumulation in SKOV-3 and T47D cells. Expression of SLC7A11 and glutathione peroxidase 4 (GPX4) was repressed by lidocaine in SKOV-3 and T47D cells. Data suggest that lidocaine, through the accumulation of Fe^2+^, iron and lipid reactive oxygen species (ROS), induces ferroptosis of ovarian and breast cancer cells, which inhibits the proliferation of these cells [114]. Using the example of olaparib for the treatment of ovarian cancer, Ting Hong et. shows a correlation between PARP inhibitor action and ferroptosis, in which ferroptosis is thought to be responsible for olaparib’s efficacy. PARP inhibition with the pharmaceutical or genetic deletion of PARP reduces the expression of the cysteine transporter SLC7A11 in a p53-dependent manner. As a result, glutathione biosynthesis is reduced, which promotes lipid peroxidation and ferroptosis. This study leads to the conclusion that stimulation of the ferroptosis process by ferroptosis inducers (FINs) will sensitise ovarian cancer cells with a mutation of the BRCA gene to this process resulting in reduced tumor proliferation [115] (Figure 2).

**Figure 3:**
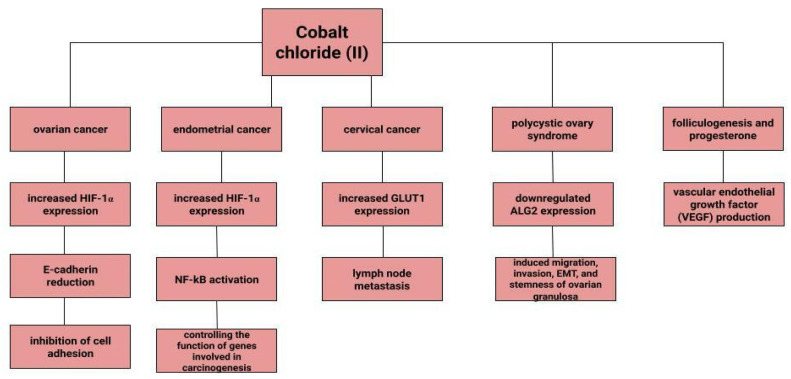
The role of cobalt chloride (II) in gynecological diseases.

Dingx Li et. investigated the function of glutathione peroxidase 4 (GPX4) in ovarian cancer cells and mouse xenografts, as a critical regulator of ferroptosis. The study was conducted in which human ovarian cancer cells and healthy ovarian epithelial cells were administered deferoxamine (DFO) and ferrous ammonium citrate (FAC). DFO chelating intracellular iron impaired ovarian cancer cell survival. In contrast, FAC and blocking GPX4 resulted in inhibition of tumor growth and induction of ferroptosis. This accelerated cell apoptosis and reduced Fe^3+^ accumulation in mouse cells [116]. Some metals, including cobalt, can affect the human ovarian cancer G1 protein-coupled receptor (hOGR1) [117]. Cobalt, through activation of mammalian ovarian G-protein-coupled receptor 1 OGR1, can induce IL-6 cytokine secretion [118]. Human ovarian cancer G-protein-coupled receptor 1 (hOGR1) and GPR4 (hGPR4) can bind to extracellular protons, leading to activation of intracellular signalling pathways through G proteins. A reduction in angiogenesis and tumor growth was observed in GPR-null mice. It is possible that it is cobalt that stimulates the zOGR1-mediated SRE-promoter. Iron was also tested, and no such relationship was found for cobalt [117]. In contrast, CoCl_2_ inducing HIF-1alpha in a time- and dose-dependent manner may lead to inhibition of cell adhesion (Figure 3), and the relationship between proliferation and invasion of HO-8910PM human ovarian cancer cells is altered by induction of hypoxia conditions and subsequent reoxysgenation [86]. The role of HIF-1α, which cobalt chloride is used to induce, in ovarian cancer cells may involve a reduction in E-cadherin, as demonstrated in two human ovarian cancer cell lines (SKOV3 and OVCAR5) [119]. The role of cobalt in the development of ovarian cancer needs to be verified in further studies.

### 4.2. Endometrial Cancer

Endometrial cancer is a neoplasm that is more likely to manifest itself while still at an early stage than ovarian cancer [17]. Abnormal uterine bleeding, which includes prolonged menstrual bleeding and acyclic bleeding, is the most common symptom of endometrial cancer [18]. Risk factors for ovarian cancer include obesity, childlessness, smoking and diabetes. There are well-recognised protective factors such as pregnancy, hormonal contraception [120]. The expression of hypoxia-inducible factor (HIF-1α), the concentration of which increases in the presence of cobalt (II) chloride [80], may correlate with endometrial cancer malignancy. The classical nuclear transcription factor NF-kB pathway is thought to be activated in endometrial cancer cells via HIF-1α [82]. Nuclear transcription factor-kB is composed of subunits of Rel family proteins (p50, p52, p65 (RelA), c-Rel and RelB) [121]. NF-kB can control the function of many genes that are involved in inflammation, apoptosis and also carcinogenesis [122]. According to some sources, the use of iron supplements could be a potential marker of endometrial cancer rather than its aetiological factor [123]. Higher iron concentrations are observed in cancerous endometrial tissues than in non-cancerous tissues [112]. Some sources indicate a positive association between heme iron intake, total iron and liver intakes and endometrial cancer risk, with no association between intake of other red processed meat [124]. However, it is likely that dietary iron, as well as iron taken with red meat, or hem iron or non-heem iron do not affect the increased risk of endometrial cancer [125].

### 4.3. Cervical Cancer

Cervical cancer remains a significant oncological challenge, and particularly so in countries where human papillomavirus (HPV) vaccines are not used, which should be used before exposure to the virus [126,127,128]. However, HPV is not the only risk factor for cervical cancer development, and other variables may influence tumor progression. In a study by Cheng et al. under CoCl_2_-induced hypoxia, the expression of glucose transporter protein 1 (GLUT1) increases progressively according to the clinical stage of cervical cancer. Correlations of increased GLUT1 expression with lymph node metastasis are observed. In normal tissue, this expression is lowest, while it is higher in cervical intraepithelial neoplasia and highest in cervical cancer [129]. H. Cunzhi, on the basis of a study of 30 patients with myoma of the uterus, 40 patients with cervical cancer, and 50 healthy patients, showed that iron levels in cervical cancer tissue were higher than in unchanged tissue, and that serum iron levels were lower in patients with cervical cancer than in healthy patients. H. Cunzhi, based on a study of 30 patients with myoma, 40 patients with cervical cancer, and 50 healthy patients, showed that iron levels are higher in cervical cancer tissue than in myoma [130].

### 4.4. Uterine Myoma

**Figure 4:**
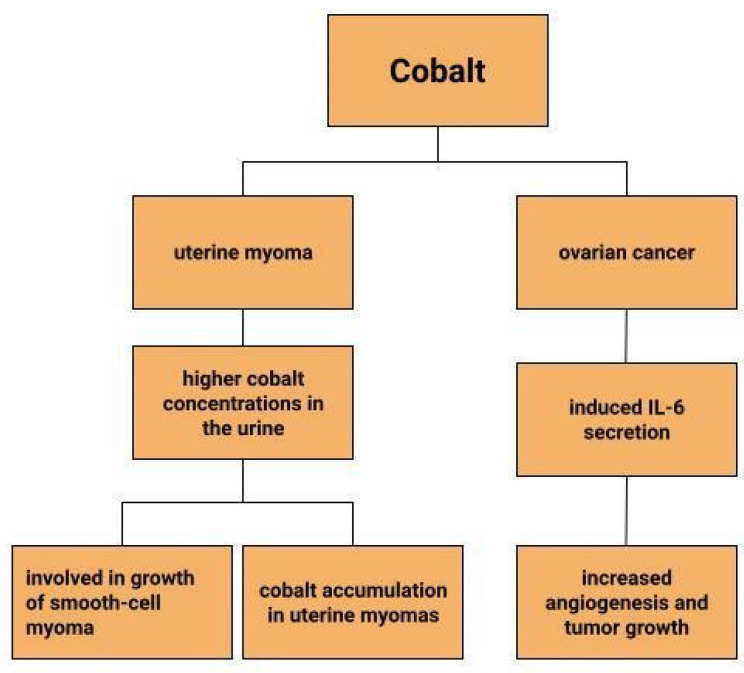
The role of cobalt in gynecological diseases.

Uterine myomas are among the most common benign uterine neoplasms [131]. Although the course is asymptomatic in most cases [132], they can sometimes cause prolonged, heavy, acyclic bleeding [133] resulting in the development of anaemia in patients with uterine myomas [134,135]. They also increase the risk of infertility, miscarriage, intrauterine growth retardation and preterm birth [136]. Depending on their location, they are divided into submucosal, intramural and subserosal [137]. A reflection of the lower serum iron concentrations in patients with myomas can be seen in measurements of iron concentrations in myometrial tissues. Compared to normal uterine tissues and cancerous uterine tissues, iron concentrations in myomas are significantly lower. Moreover, compared to other trace metals, it is the iron concentrations that show greater tissue-specific differences [135]. Similar observations are made by H. Cunzhi et al. who, on the basis of a study of 30 patients with myoma, 40 patients with cervical cancer, and 50 healthy patients, showed that iron concentrations were higher in cervical cancer tissue than in myoma [130]. There are attempts to look for a link between trace elements and uterine myomas. One study found higher cobalt concentrations in the urine of patients with uterine myomas, which is explained by the authors in two ways (Figure 4). One would be that higher cobalt exposure may contribute to the growth of smooth-cell myoma. A second reason considered for the increased cobalt levels in women with uterine myomas compared to women without this benign tumor could be that uterine myomas are a site of cobalt accumulation [138]. However, this relationship requires further research.

### 4.5. Endometriosis

Endometriosis is a chronic disease in which active endometrial tissue is found outside the uterine cavity [139]. Patients experience increased lower abdominal pain, heavy menstrual bleeding, and these women are also diagnosed with infertility [140]. Retrograde menstruation, chorionic metaplasia and residual Mϋllerian duct are mentioned in the pathogenesis. In addition, genetic factors as well as environmental factors may influence the development of endometriosis, resulting in the development of chronic inflammation [141]. In relation to the effect of iron on inflammation through the induction of oxidative stress in the cell, the role of iron in endometriosis is being investigated.

Through the high iron content of endometrial cysts, low-oxygen conditions may occur, leading to destabilisation of iron regulatory protein 2 (IRP2) resulting in too many iron (II) ions entering the stroma cells. Such a condition may be a potential factor for carcinogenesis in endometriosis [142]. In a study by Mori et al., it is suspected that ectopic endometrial tissue provides protection to ovarian epithelial cells by taking up excess iron. It was shown that the concentration of catalytic Fe (II) was higher in ectopic endometrial stromal cells than in normal eutopic endometrial stromal cells [143]. Endometriosis is among the factors in the development of ovarian clear cell adenocarcinoma and endometrioid carcinoma and are therefore endometriosis-associated ovarian cancers (EAOCs) [144]. Oxidative stress has been linked to the inflammatory response in endometriosis, and this may be iron-dependent [145,146]. Akashi et al. observed increased iron in ovarian endometrioma (OE) and clear cell carcinoma (CCC). They presented the localisation of iron in epithelial cells and in the ovarian stroma and that macrophages are involved in iron deposition in these cells, more specifically under the epithelium of ovarian endometrioma. These are the macrophage markers CD11c, CD163, CD206, and more CD163+ and CD206+ cells are observed in the deeper layer of the OE stroma than CD11c+ cells. Similarly, for CCC, in whose stroma CD163+ and CD206+ cells are found. The expression of iron transport proteins in OE and CCC, such as divalent metal transporter 1 (DMT1), transferrin receptor (TfR) and ferroportin FPN, is high, with TfR and FPN expression at a higher level in CCC than in OE [144].

### 4.6. Polycystic Ovary Syndrome

Polycystic ovary syndrome (PCOS) is one of the most common endocrinopathies among young women [147]. PCOS consists of hyperandrogenemia, ovulatory disorders, hyperinsulinism, and insulin resistance [148]. In addition, the syndrome raises the risk of endometrial cancer [20], as well as diabetes, cardiovascular disease [149] and contributes to reduced female fertility [150]. The role of iron in PCOS appears to be important, if only because iron may have a role as an oxidative factor in the development of insulin resistance in these patients [151]. Some studies suggest that this reduction in iron concentration may participate in the pathophysiology of polycystic ovary syndrome [152]. Other observations point to the possible efficacy of lowering iron levels in improving glucose tolerance in PCOS patients, as it has been noted that Fe is elevated in PCOS patients and especially when this syndrome is accompanied by abnormal glucose tolerance, suggesting that excess iron may be one of the causes of insulin resistance and diabetes in PCOS patients [153]. Insulin contributes to increased intestinal iron absorption and iron deposition in tissues. The findings of Luque-Ramírez et al. present that it is hyperinsulinemia and insulin resistance that cause increased tissue ferritin levels in women with PCOS [154]. Furthermore, it is possible that when insulin resistance is present, reduced hepcidin concentrations occur, resulting in increased iron levels [155]. The association of hepcidin and iron levels in PCOS patients has not been confirmed in another study, suggesting the need for expanded research [151]. Only one paper reports on the association between cobalt and PCOS. Serum concentrations of magnesium, cadmium and also cobalt in patients with PCOS were shown to differ between the study group and the control group [156]. The relationship between α-1,3/1,6 mannosyltransferase (ALG2) and ovulation is being investigated in patients with polycystic ovary syndrome. CoCl_2_ may downregulate ALG2 expression in PCOS patients, which may induce migration, invasion, EMT, and stemness of ovarian granulosa cells. In contrast, elevated ALG2 levels are observed in the serum of PCOS patients, which is being considered as a biomarker for the diagnosis of PCOS [157].

### 4.7. Folliculogenesis and Progesterone

Deficiency of vitamin B12, which contains cobalt, can affect oestrogen levels, stopping ovulation [158]. In animals, cobalt deficiency results in lower conception rates [159]. In addition, cobalt, as a probable factor affecting the secretory activity of ovarian granulosa cells, would be expected to affect progesterone and insulin-like growth factor 1 (IGF-1) concentrations [160]. Kolesarova et. Conducting a study on porcine ovarian granulosa cells, that the release of IGF-1 by these cells varies according to the concentration of cobalt administered. A similar effect was also observed on rat ovarian cells, in which the value of released IGF-I when administered 90 μg.mL of cobalt sulfate was 18.64 +/− ng.mL, against 8.85 +/− 2.99 ng.mL in the control group, i.e., without cobalt supply. In contrast, progesterone release by granulosa cells was inhibited by cobalt at a concentration of 0.09 mg/mL, but no such relationship was shown at higher cobalt concentrations [160]. Using rat ovarian cells as an example, it was noted that cobalt in the form of cobalt sulfate (CoSO_4_·7H_2_O) at doses of 170–500 µg mL can inhibit progesterone secretion [161]. In contrast, a study by Grasselli F. et al. does not link the inhibition of progesterone production to CoCl_2_ but is involved in vascular endothelial growth factor (VEGF) production in granulosa cells, thus controlling the folicular angiogenic process [162]. Bax and caspase-3 are among the factors involved in apoptosis. There are associations of cobalt with decreased expression of Bax and caspase-3 in rat ovarian cells [161]. The direct effect of ions including Co^2+^ on embryogenesis was tested by culturing mouse blastocysts in the presence of individual ions in vitro. It was observed that 100 microM cobalt ions reduced trophoblast size. Cobalt ions would also be expected to affect morphological changes including a reduction in adhesion capacity [163]. The above data suggest that cobalt or its compounds may affect folliculogenesis.

### 4.8. Placental Hypoxia

**Figure 5:**
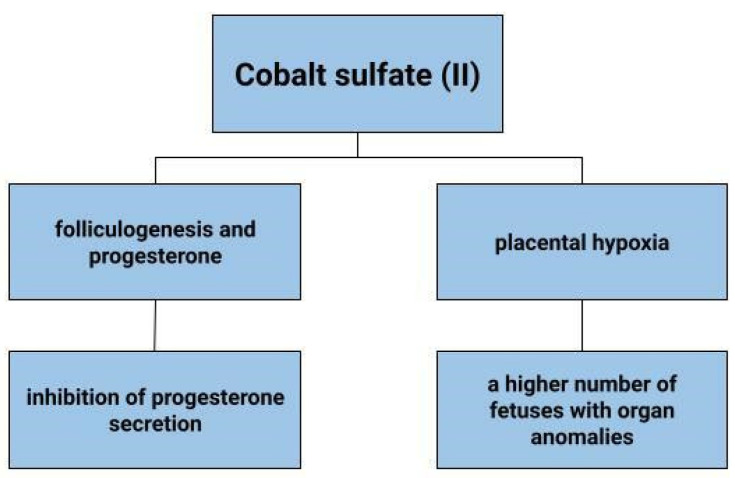
The role of cobalt sulfate (II) in gynecological diseases.

Hypoxia can adversely affect the development of the placenta. It can result in reduced fetal growth, as well as pre-eclampsia. In a state of cellular hypoxia, which in the study by Baumann et al. was induced by desferroxamine and cobalt chloride, there was an increase in the expression of the glucose transporter 1 GLUT1 in the human placental cell line that originates from a choriocarcinoma (BeWo) [164]. Furthermore, the increase in GLUT was shown to be due not only to low oxygen concentration, but also to other factors that induce HIF-1α. These results depict that hypoxia increases glucose transport into cells [164]. The effects of cobalt sulphate on pregnant mice and fetuses have also been tested (Figure 5). Cobalt can enter the blood and amniotic fluid of the foetus. Dose-dependent toxicity of this element was therefore observed in both mother and fetus. A higher number of fetuses with delayed skeletal development and weight were found, as well as organ anomalies, such as kidney, eye, genitourinary system [165]. In contrast, no toxic effects of cobalt on fetuses were observed in the work of Paternain et al. [166].

**Table 1:** Effects of cobalt and its compounds on specific tissues.

Tissues	Cobalt Form	Result	Reference
Ovary Animal tissue	Cobalt (II) chloride	Probably activated the zOGR1-mediated signaling pathway à increase in angiogenesis, increase in tumor size	Negishi et al. [117]
Ovary Gilts tissue	Cobalt (II) sulfate	Increase in released IGF-1, Inhibition of progesterone release	Kolesarova et al. [160]
Ovary Rat tissue	Cobalt (II) sulfate	Inhibition of progesterone release	Roychoudhury et al. [161]
Granulosa cells Human tissue	Cobalt (II) chloride	No effect of CoCl_2_ on progesterone, Involvement in the foliculalar angiogenic process through stimulation of VEGF production	Grasselli et al. [162]
Ovary Rat cells	Cobalt (II) sulfate	Decreased expression of Bax and caspase-3 à inhibition of apoptosis	Roychoudhury et al. [161]
Endometrial cancer	Cobalt (II) chloride	Effects on the classical nuclear pathway of the transcription factor NF-kB via HIF-1α; effects on carcinogenesis	Yoshida et al. [82]
Choriocarcinoma cells, a trophoblast cell model, and human placental villous tissue explants	Cobalt (II) chloride	Placental hypoxia	Baumann et al. [164]
Pregnant mice, fetuses	Cobalt (II) sulfate	Increased number of fetuses with delayed skeletal development and weight, organ anomalies-kidneys, eyes, genitourinary system	Szakmáry et al. [165]
Trophoblast, Mice	Cobalt (2+)	Reduction in trophoblost size and adhesion capacity	Paksy et al. [163]
Uterine myomas	Cobalt (2+)	Influence on smooth cell myxoma growth or myxoma as a site of cobalt accumulation	Johnstone et al. [138]
Polycystic ovary syndrome	Cobalt (2+)	No relation	Kurdoglu et al. [156]

zOGR1-zebrafish ovarian cancer G-protein-coupled receptor 1, IGF-1-insulin-like growth factor 1, CoCl_2_-cobalt (II) chloride, VEGF-vascular endothelial growth factor, NF-kB-nuclear factor kappa-light-chain-enhancer of activated B cells, HIF-1α-hypoxia-inducible factor 1-alpha.

## 5. Conclusions

Cobalt and iron, through their properties, compound formation and participation in a number of chemical reactions in the human body, should not be interpreted without insight into these mechanisms. A review of the literature shows that cobalt compounds may be factors that increase the risk of cancer development and its faster progression. However, cobalt ions alone do not appear to pose such a risk. Most studies are based on animal cells, investigating the effects of cobalt (II) chloride. Cobalt salts can prevent DNA repair, contributing to the development of mutagenic agents and, through these processes, lead to cancer development. In addition, cobalt binding HIF-1α leads to the development of hypoxia-like conditions. However, due to its physical properties, it can be used in the radiotherapy of gynecological cancers, especially in countries with relatively lower budgets for cancer therapy. Most of the information relates to animal cells, which allows cobalt compounds to be considered as potentially carcinogenic and allocated to the appropriate group, as obtained in the IARC analysis. However, more evidence of the effect of cobalt on the development of gynecological diseases in humans is lacking. Iron may contribute to cancer and other disorders in gynecology. It has been observed at higher concentrations in cervical cancer cells, endometrial cancer, and ovarian cancer compared to healthy tissue. The role of iron in the development of polycystic ovary syndrome remains particularly unclear, and it is possible to observe lower iron concentrations in myomas than in non-transformed uterine tissues, but this may be related to heavy bleeding in these patients. Due to the participation of cobalt and iron in enzymatic, redox reactions, in which reactive oxygen species are formed, the existence of corresponding levels of these elements in the human body cannot be ruled out.

## Data Availability

Not applicable.
